# Enhanced Electrochromic Smart Windows Based on Supramolecular Viologen Tweezers

**DOI:** 10.1021/acs.chemmater.4c03174

**Published:** 2025-03-12

**Authors:** Jaume Ramon Otaegui, Silvia Mena, Jovelt M. Dorsainvil, Gonzalo Guirado, Daniel Ruiz-Molina, Jordi Hernando, Jonathan C. Barnes, Claudio Roscini

**Affiliations:** † Departament de Química, Universitat Autònoma de Barcelona, Edifici C/n Campus UAB, Cerdanyola del Vallès 08193, Spain; ‡ Instituto de Microelectrónica de Barcelona (IMB-CNM, CSIC), Cerdanyola del Vallès 08193, Spain; § Department of Chemistry, 7548 Washington University in St. Louis, St. Louis, Missouri 63130, United States; ∥ Catalan Institute of Nanoscience and Nanotechnology (ICN2) CSIC and BIST, Campus UAB, Bellaterra, Barcelona 08193, Spain

## Abstract

Extending the spectral response of viologen-based electrochromic devices to the near-infrared region is essential to enhance their performance for smart window applications. Although synthetic and formulation modifications have been proposed to achieve this goal, these strategies always come at the expense of deteriorating the electrochromic behavior of the system. To overcome this limitation, herein we exploited the supramolecular chemistry of viologen molecular tweezers, which undergo an intramolecular dimerization process upon reduction that leads to broad light absorption through the visible and near-infrared spectra. We observed this behavior to take place at low concentrations in a variety of electrolytic media, including solid-state ionogels that could be applied to the fabrication of electrochromic devices. Better spectral response, lower operation voltage, and higher stability were measured for these devices relative to analogous systems based on viologen monomers. As a result, when used as electrochromic smart windows, the viologen tweezer-based devices exhibited enhanced modulation of solar heat gain with reduced energy consumption, thereby demonstrating the potential of viologen supramolecular chemistry to rationally improve the performance of electrochromic devices.

## Graphical abstract

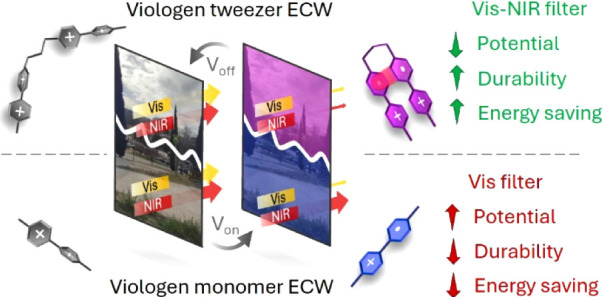

## 1 Introduction

By dynamically regulating sunlight transmission through fenestration elements, smart windows (SW) are one of the pivotal strategies toward more sustainable buildings, which currently account for about 40% of world’s energy consumption and 30% of annual greenhouse emissions.^1–6^ In addition, SWs aid in increasing occupant’s comfort and privacy in buildings, while also finding application in other areas where the control of solar irradiation is required (e.g., in greenhouses^7^). Among the different technologies proposed to accomplish these objectives,^8–10^ electrochromic SWs (ECW) that reversibly switch between colorless and colored states upon application of external voltages are of special importance, and they have already reached the market.^11–14^ However, current commercial ECWs, which are mainly based on inorganic electrochromic materials such as tungsten trioxide (WO_3_), suffer from some limitations that hamper their broad application—e.g., high cost, difficult processing and poor color tunability.^13,15^ In light of this, novel all-organic electrochromic materials should be developed to surpass these hurdles and further expand the use of ECWs.

*N*,*N*′-Disubstituted-4,4′-bipyridinium derivatives, commonly known as viologens (V^2+^), are one of the most promising nonexpensive alternatives to inorganic electrochromic materials.^16–19^ Normally, viologen solutions are colorless, and they become reversibly colored upon a one-electron reduction to their radical cation form (V^•+^), thus absorbing sunlight. For ECW fabrication, viologens offer numerous advantages, such as facile preparation, easy color tunability by synthetic modification (from blue to green, purple and even dark gray),^20–23^ excellent stability,^24–29^ and very strong coloration under low redox potentials, exhibiting extinction coefficients that surpass those of typical inorganic compounds.^30,31^ Despite these advantageous features, it must be noted that the absorption of the colored V^•+^ viologen form is normally restricted to the ultraviolet (UV) and visible ranges, which barely account for 50% of sunlight spectrum.^2^ As a result, the energy-saving efficiency of viologen-based ECWs is limited because they cannot efficiently modulate solar irradiation in the near-infrared (NIR) region.^2,20–24,26,32,33^ Although NIR-absorbing viologen radical cations have been successfully synthesized by extending the electronic conjugation in their pyridinium moieties, this comes at the expense of some important downsides for ECW applications: (1) a significant loss of the initial transparency and the overall color contrast, as the corresponding V^2+^ dicationic form becomes yellow; and (2) an increase of the reduction potential and, therefore, of the device energy consumption.^34–37^

To overcome these drawbacks while expanding the response of viologen-based ECWs to the NIR region, the viologen-based radical molecular recognition could be exploited. First described in the 60s,^38–40^ pairs of V^•+^ species tend to form supramolecular stacks via radical pairing that overcomes charge repulsion.^41^ Interestingly, the formation of these viologen π-dimers (also known as pimers)^41,42^ produces an extended electronic conjugation over the two paired bipyridinium rings of the interacting V^•+^ units.^43–45^ As a result, they exhibit an additional low-energy absorption band relative to the individual viologen radical cations, which ranges from 800 to 1400 nm depending on the degree of pimerization. Consequently, this makes the electrochromic response of viologens develop into the NIR region, while preserving a colorless state in the nonreduced form as the two initial viologen units only absorb in the UV region.^41,46^ However, for this concept to be exploited in ECWs, high viologen concentrations are required to ensure spontaneous pimer formation, which has been associated with poor operational stability due to precipitation and consequent loss of redox activity, among others.^23,28^ In addition, intermolecular pimer formation is very sensitive to solvent polarity and only occurs under certain conditions,^46^ which makes it even harder to be used as a general strategy to improve ECWs spectral sensitivity.

Herein we propose an alternative method to capitalize on viologen pimerization for broadening the optical response of viologen-based ECWs without detrimentally affecting their performance (Figure 1). Our approach consists in favoring pimer generation at low viologen concentrations through a rational design: the formation of intramolecular dimers in constructs where two viologen units are bonded through a flexible linker—the so-called viologen molecular tweezers.^42,47–50^ Indeed, these types of compounds have been widely exploited in supramolecular chemistry for the preparation of molecular machines^51–53^ and mechanical actuators,^54–56^ among other stimuli-responsive systems.^57,58^ To pioneer their use to the fabrication of enhanced ECWs, in this work we selected one of the simplest cases of viologen tweezers reported as a benchmark system: a propyl-bridged viologen dimer, which is known to undergo a reversible redox-induced transition from a fully colorless oxidized state ((2V)^4+^) to a highly colored pimer form absorbing both in the visible and NIR regions upon a one-electron reduction of each bispyridinium unit ((2V)^2•+^).^47,49,59^ In addition, owing to the enhanced stability of the pimer (2V)^2•+^ caused by the radical molecular recognition between the paired viologen units, the first electronic reduction of the colorless form (2V)^4+^ takes place at lower potentials than for analogous monomeric viologens.^59–61^ As a result, its use as electrochromic material in ECWs could also benefit from a reduced energy consumption over conventional viologen-based devices. To demonstrate these advantageous features of viologen molecular tweezers for the fabrication of ECWs, we used polymer ionogels as electrolyte media for the redox-induced operation of (2V)^4+^, which are known to allow the preparation of all-in-one electrochromic devices with simplified architectures.^23,62,63^

**Figure 1:**
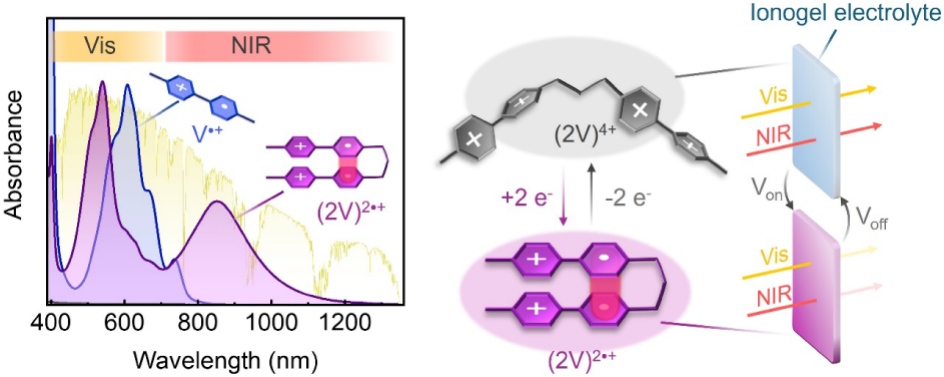
Upon reduction of viologen tweezers, intramolecular radical pairing causes absorption to extend deep into the NIR region ((2V)^2•+^), thus better covering the solar irradiance spectrum compared to reduced viologen monomers (V^•+^). By introducing these dimers into ionogels, we aim to fabricate ECWs with enhanced energy-saving performance by switching between fully transparent and visible-NIR blocking states.

## 2 Results and Discussion

### 2.1 Viologen Pimerization in Ionogels

Viologen dimers linked through flexible tethers such as alkyl chains can undergo intramolecular radical pairing—i.e., pimer formation—upon a one-electron reduction of both viologen units.^47^ However, this phenomenon is strongly influenced by linker length. For ethyl spacers, intramolecular pimer formation is inhibited due to the high torsion angle required to ensure π–π stacking of the viologen units.^61^ In contrast, pimerization can occur with longer alkyl chain linkers, and it is the most favored for propyl tethers, as demonstrated by a variety of experimental techniques.^42,48,49,59,60,64–66^ In light of this, 1,1′-(propane-1,3-diyl)­bis­(1-methyl-[4,4′-bipyridine]-1,1′-diium) hexafluorophosphate, a propyl-bridged viologen dimer salt (2V-4PF_6_), was prepared according to literature procedures (Figure 2a).^82^ When dissolved in a common organic solvent such as acetonitrile, this compound exhibited the expected electrochromic behavior: while (2V)^4+^ only absorbed in the UV region in its initial tetracationic state, two additional absorption bands appeared at λ_abs_^max^ = 534 and 835 nm upon two one-electron reductions that can be ascribed to intramolecular pimer formation ((2V)^2•+^, Figure 2b and Table S1). As a result, the sample turned from colorless to violet. On the contrary, when a reference methyl viologen monomer (Figure 2a, V^2+^) was reduced under the same conditions, only the characteristic absorption band of its radical form (V^•+^) at λ_abs_^max^ = 608 nm was observed, thus producing blue coloration and indicating the lack of intermolecular radical pairing (Figure 2b and Table S1). As anticipated, pimerization also caused a clear difference in the redox potentials measured for (2V)^4+^ and V^2+^ in acetonitrile by cyclic voltammetry (Figure 2c and Table S2). Although both compounds showed the two reversible cathodic waves that are characteristic of viologens, the additional stabilization energy induced by pimer formation favored the first reduction of (2V)^4+^ (*E*_(2V)^4+^/(2V)^2•+^_^0^ = −0.334 V vs Ag/AgCl) over V^2+^ (*E*_V^2+^/V^•+^_^0^ = −0.449 V vs Ag/AgCl), while it retarded the subsequent reduction to the yellow-colored neutral state (*E*_(2V)^2•+^/2V_^0^ = −0.901 V vs Ag/AgCl and *E*_V^•+^/V_^0^ = −0.865 V vs Ag/AgCl). As a result, the redox-induced coloration of the viologen dimer could be triggered at lower potentials (Δ*E*_(2V)^4+^–V^2+^_^0^ = 0.115 V).

**Figure 2:**
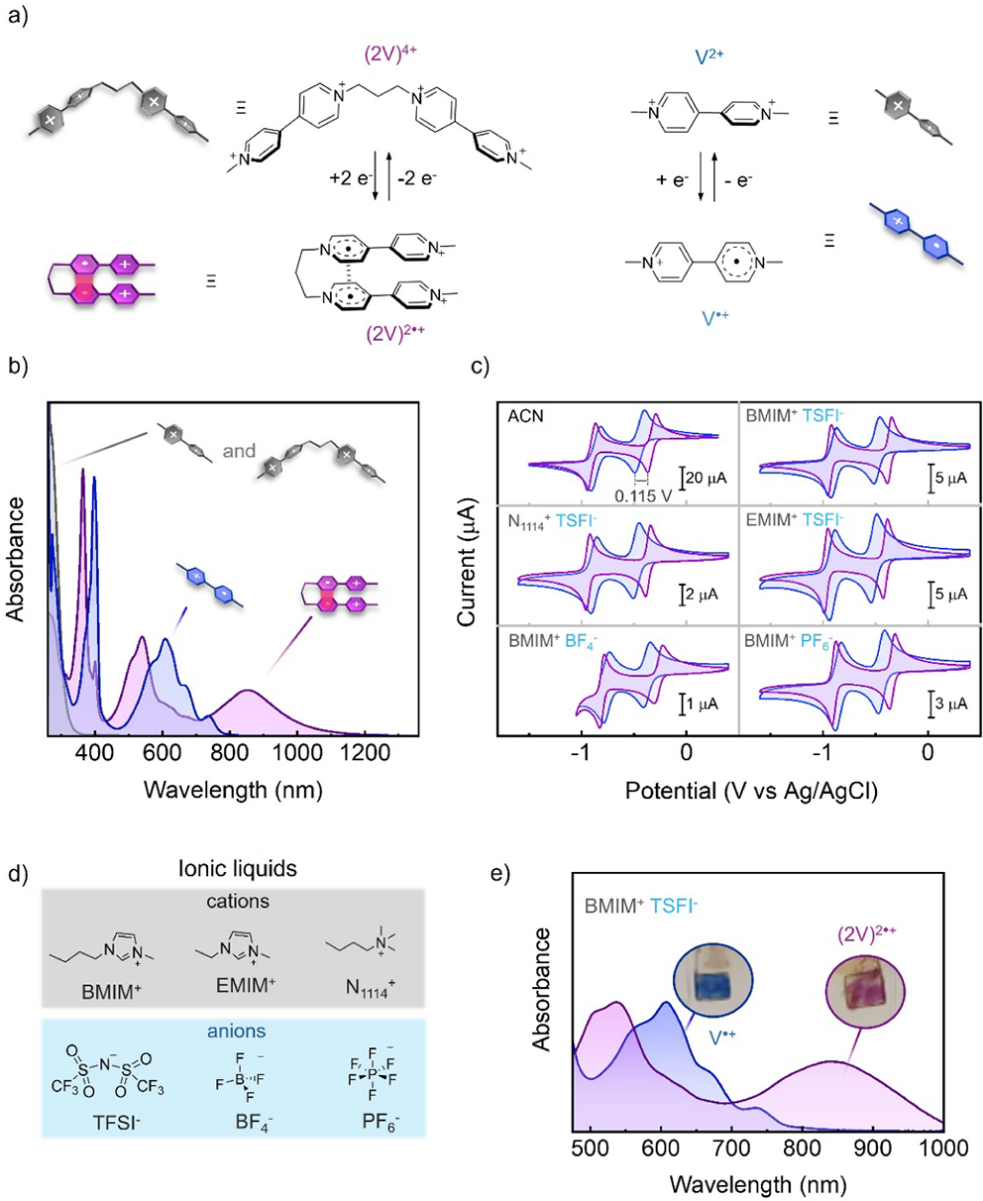
(a) Molecular structures of (left) dimer (2V)^4+^ and the pimer formed upon one-electron reduction of each of the viologen units, and (right) monomer V^2+^ and the radical cation obtained upon a one-electron reduction. (b) Absorption spectra of (2V)^4+^ (gray), V^2+^ (gray), (2V)^2•+^ (purple) and V^•+^ (blue) in acetonitrile. The reduced species were obtained using Zn powder as a reducing agent (see the Supporting Information for more details). Notice that (2V)^4+^ and V^2+^ exhibit the same absorption band, entirely placed in the UV region. (c) Cyclic voltammogram of solutions of (2V)^4+^ (purple, *c* = 5 mM) and V^2+^ (blue, *c* = 10 mM) in ACN/0.1 M TBA PF_6_ and several ILs (working electrode: glassy carbon; counter electrode: Pt; reference electrode: Ag/AgCl; scan rate: 0.1 V·s^–1^). (d) Molecular structures of the cations and anions of the ILs utilized. (e) Absorption spectra of (2V)^2•+^ (purple) and V^•+^ (blue) in [BMIM]­[TFSI] obtained from initial (2V)^4+^ and V^2+^ solutions (*c* = 5 and 10 mM, respectively) under an applied potential of *E*_app_ = −0.75 V (vs Ag/AgCl). The absorption spectra in (b) and (e) have been normalized to the absorption maximum of (2V)^2•+^ and V^•+^ in the visible range.

Interestingly, the electrochromic behavior of (2V)^4+^ (and of reference viologen V^2+^) was preserved in ionic liquids (ILs), an alternative to organic solvent-based liquid electrolytes that offers multiple advantages for the preparation of electrochemical devices.^23,62,63^ Thus, pimerization was also found to selectively occur for (2V)^4+^ in 1-butyl-3-methylimidazolium bis­(trifluoromethylsulfonyl)­imide ([BMIM]­[TFSI]), which led to an even lower reduction potential compared to that used for V^2+^ (Δ*E*_(2V)^4+^–V^2+^_^0^ = 0.121 V) and a broad absorption for its dicationic state (2V)^2•+^ extending along the visible and NIR regions (λ_abs_^max^ = 538 and 840 nm) (Figure 2c–e, Tables S1–S2). By contrast, lower current values were measured for the reduction of (2V)^4+^ and V^2+^ in [BMIM]­[TFSI], a common phenomenon when using ILs as electrolytes due to their higher viscosity that slows down the molecular diffusion of the electroactive species toward the electrodes.^67,68^ Similar features were registered in other common commercially available ILs containing different anions and cations, such as 1-ethyl-3-methylimidazolium bis­(trifluoromethylsulfonyl)­imide ([EMIM]­[TFSI]), butyltrimethylammonium bis­(trifluoromethylsulfonyl)­imide ([N_1114_]­[TFSI]), 1-butyl-3-methylimidazolium tetrafluoroborate ([BMIM]­[BF_4_]), and 1-butyl-3-methylimidazolium hexafluorophosphate ([BMIM]­[PF_6_]) (Figure 2c,d and Table S2). Overall, these results demonstrate that the enhanced electrochromic performance of viologen tweezers resulting from intramolecular radical ion pairing can be reproduced in a large variety of liquid electrolytes, in contrast to the intermolecular pimerization only observed for monomeric viologens in selected media at high concentrations.^46^

After finding evidence of pimer formation for (2V)^2•+^ in IL solutions, we investigated if this behavior could be transferred to solid electrolytes. With this aim, we incorporated our viologen compounds in polymer ionogels (IGs), which are leakage-free self-standing materials that preserve most of the advantages of liquid electrolytes such as transparency and high ionic conductivity.^23,62,63^ Based on our previous work on electrochromic IGs,^69–71^ we used poly­(vinylidene fluoride-*co*-hexafluoropropylene) (P­(VDF-*co*-HFP)) as the polymer phase of the gel where IL solutions of (2V)^4+^ (and V^2+^) were immobilized by solvent evaporation (Figure 3a). After some preliminary experiments, we selected [BMIM]­[TFSI] as a IL because of its reduced cost and optimal performance. Moreover, we added LiTFSI as a salt, which has been reported to increase ion mobility and ionic conductivity when dissolved in ILs at adequate concentrations,^72,73^ thus enhancing the electrochromic properties of the final IGs. The obtained ionogels (thickness ∼250 μm) were flexible and highly transparent (%*T* > 80% in the visible region), and exhibited an electrical conductivity around 0.170–0.200 mS cm^–1^ (Figures 3b, S1–S2 and Table S3).

**Figure 3:**
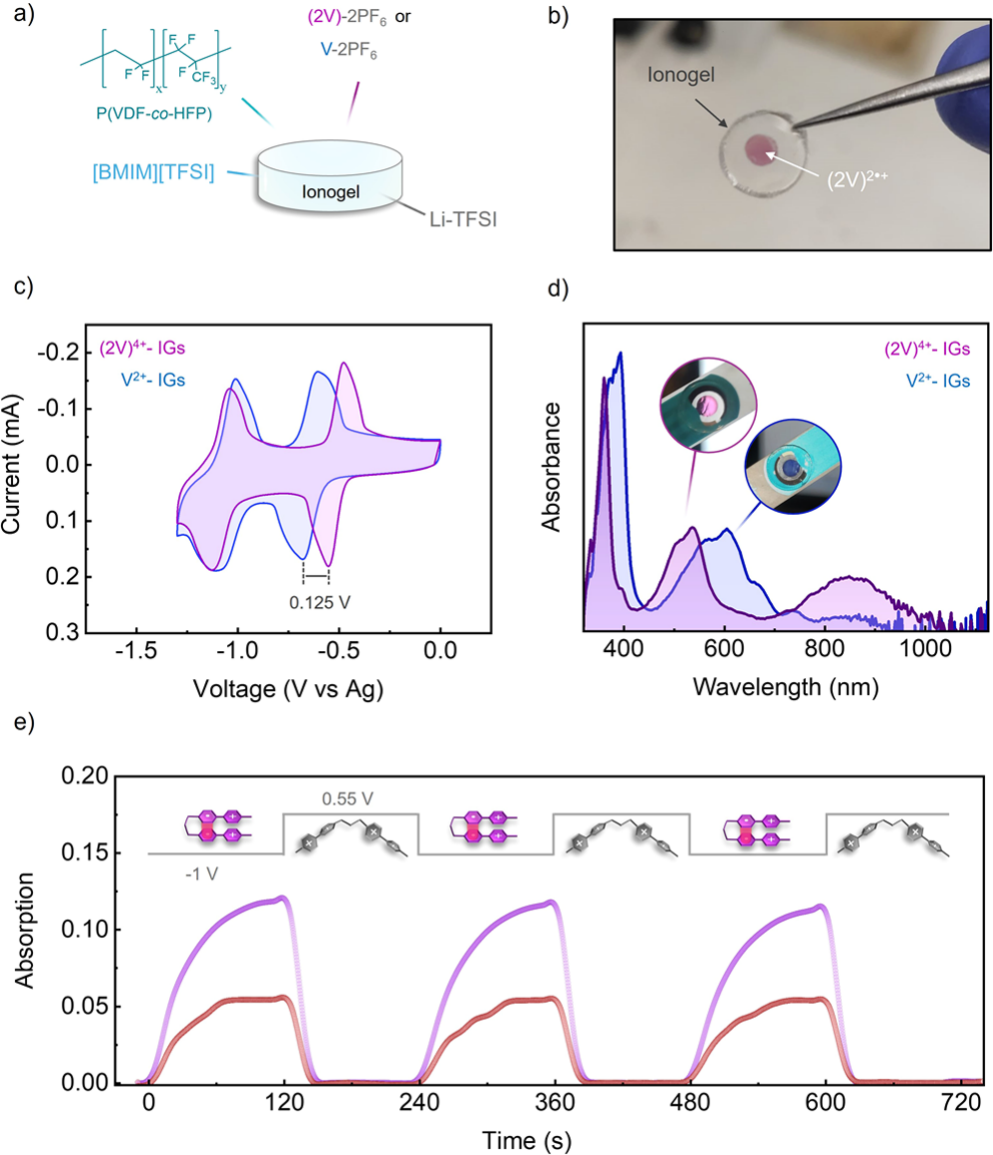
(a) Schematic diagram of the composition of the electrochromic ionogels developed in this work. (b) Image of a free-standing (2V)^4+^-doped IG right after applying a reductive potential of *E*_app_ = −1.0 V (vs Ag) for 60 s, which caused violet coloration in the central region that was in contact with the working graphite electrode. (c) Cyclic voltammograms of the (2V)^4+^- (purple) and V^2+^-loaded (blue) IGs (0.15 V s^–1^, working electrode: graphite; pseudoreference electrode: Ag). (d) Absorption spectra of the IGs after applying *E*_app_ = −1.2 V (vs Ag) for 60 s. These spectra were normalized to the absorption maximum of (2V)^2•+^- (purple) and V^2+^-loaded (blue) IGs in the visible region for comparison purposes. The insets show the actual color of the IGs in contact with the ITO working electrode. (e) Variation of the absorbance at λ = 534 (purple) and 845 nm (red) of a (2V)^4+^-loaded IG during three consecutive reduction (*E*_app_ = −1.0 V (vs Ag)) and oxidation (*E*_app_ = +0.55 V (vs Ag)) cycles (working electrode: ITO; pseudoreference electrode: Ag).

Next, we investigated the electrochromic properties of the IGs on a screen-printed three-electrode system, for which we selected the concentrations of (2V)^4+^ and V^2+^ to be 0.25 and 0.24 wt %, respectively, which correspond to the same number of viologen units in both cases. As previously found in liquid solutions, a lower potential was also measured in ionogels for the first reduction wave of (2V)^4+^ relative to monomeric V^2+^ (Δ*E*_(2V)^4+^–V^2+^_^0^ = 0.125 V), whereas the electrochemical window between the two reduction peaks increased (Figure 3c). Interestingly, in IGs the Δ*E*_(2V)^4+^–V^2+^_^0^ is similar to ILs, further demonstrating the potential advantage of the use of (2V)^4+^ as electrochromic material. In addition, upon reduction of (2V)^4+^ to (2V)^2•+^, the area of the IGs in contact with the working electrode turned violet and the two absorption bands in the visible and NIR regions that are indicative of pimer formation were measured (λ_abs_^max^ = 534 and 845 nm) (Figure 3b and d). Moreover, lowering the concentration of (2V)^4+^ to 0.042 wt % produced the same visible and NIR bands with equivalent intensity ratio upon reduction, thereby demonstrating the intramolecular nature of the pimerization process in the IGs (Figure S3). In contrast, reduction of V^2+^ to V^•+^ led to blue coloration of the ionogels and negligible absorption in the NIR spectrum, which proves that radical ion pairing did not take place for the reference viologen monomer IGs. In fact, the development of a minor NIR absorption band arising from intermolecular pimer formation could only be observed when increasing 10-fold the concentration of V^2+^ in the IGs (2.4 wt %), which also resulted in a loss of transparency in the initial material that suggests viologen aggregation and precipitation, a very undesired process in electrochromic devices (Figure S4). Therefore, these results indicate that intermolecular radical ion pairing is highly disfavored in our ionogels and further supports the intramolecular nature of the π-dimers observed for (2V)^4+^ upon reduction, which can be produced at very low electrochrome concentrations favoring operation stability. Interestingly, pimerization of (2V)^4+^ in the IGs was found to be fully reversible by applying an oxidative potential, which allowed repetitive operation of their electrochromic response without apparent degradation (Figure 3e). Finally, we found that this behavior was not exclusive for [BMIM]­[TFSI] IG but also for others based on different ILs (Figure S5).

### 2.2 Electrochromic Devices Based on Viologen Tweezers

The successful control of viologen tweezers’ pimerization in IGs motivated us to use these materials to build (2V)^4+^-based electrochromic devices ((2V)^4+^-ECD). For comparison purposes, analogous V^2+^-based ECDs were also prepared as a reference (V^2+^-ECD). In both cases, all-in-one ECDs with a sandwich-like architecture were fabricated by simply drop-casting ionogel precursor solutions onto an ITO-glass electrode and covering the sample with a second ITO-glass after solvent evaporation (Figure 4a, see the Supporting Information for further details). Typically, ∼0.2 mm thick electrochromic IG layers were obtained in this way, where the same concentrations of viologen units were used for both (2V)^4+^- and V^2+^-based devices as described above (*c*_(2V)^4+^_ = 0.25 wt % and *c*_V^2+^_ = 0.24 wt %). However, we slightly modified the formulation of the IGs employed for ECD fabrication by adding ferrocene (Fc, 0.1 wt %), which has been reported to enhance the electrochromic properties of viologen-based devices (Figure S6).^24,28^ Both types of ECDs prepared were essentially colorless and transparent at 0 V, but became colored when reductive voltages were applied. Interestingly, at *E*_app_ = −1.5 to −2.5 V, (2V)^4+^-ECDs displayed the archetypical violet coloration and NIR absorption band of the pimer, while the V^2+^-based devices essentially showed the radical cation visible absorption and the corresponding blue color (Figure 4b,c). Only at the highest reductive potential tested (*E*_app_ = −2.5 V), the buildup of a low intensity NIR absorption band could be measured for V^2+^-ECDs, which suggests the onset of intermolecular pimerization (Figure 4c). It should also be noted that, despite having the same electrochrome concentration, (2V)^4+^-ECDs developed higher visible absorption—i.e., stronger coloration—than V^2+^-ECDs at *E*_app_ = −1.5 and −2.0 V. This behavior should be ascribed to the lower reduction potential of (2V)^4+^ caused by pimer formation, as previously observed in liquid and solid electrolyte samples.

**Figure 4:**
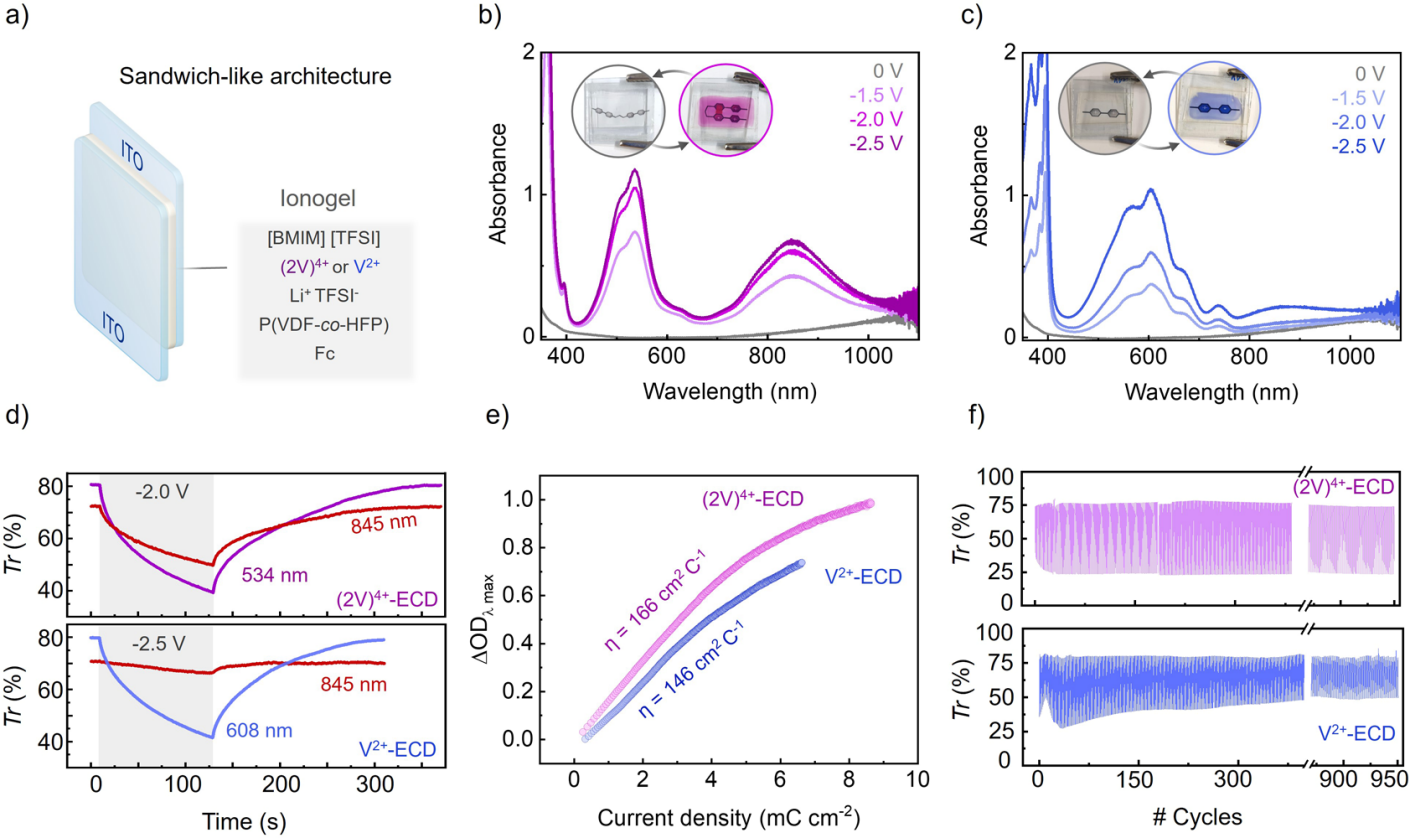
(a) Schematic diagram of ECD structure. (b,c) Absorption spectra at increasing reductive potentials of (b) (2V)^4+^-ECDs and (c) V^2+^-ECDs. Measurements were taken after 120 s of application of each voltage. The insets show images of the devices at (left) *E*_app_ = 0 V and (right) *E*_app_ = −2.0 V. (d) Transmittance (Tr (%)) variation during a coloration-bleaching cycle for (top) (2V)^4+^-ECDs and (bottom) V^2+^-ECDs. Coloration was induced by applying a reductive potential for 120 s, while bleaching took place in open circuit. Time dependent transmittance traces are shown for both visible and NIR wavelengths. (e) Coloration efficiency of (2V)^4+^-ECDs and V^2+^-ECDs in the visible region (λ_abs_ = 534 and 608 nm, respectively). (f) Coloration-bleaching cycles for (top) (2V)^4+^-ECDs and (bottom) V^2+^-ECDs. Coloration was promoted at *E*_app_ = −2.0 V ((2V)^4+^-ECD) or −2.5 V (V^2+^-ECD) for 120 s, and bleaching occurred in open circuit for 300 s. Transmittance variation is shown at λ = 534 (2V^4+^-ECD) and 608 nm (V^2+^-ECD).

The (2V)^4+^-based ECDs exhibited relatively fast coloration kinetics, achieving around −40% and −25% transmittance modulation (ΔTr) in the visible (λ_abs_ = 534 nm) and NIR regions (λ_abs_ = 845 nm), respectively, by applying *E*_app_ = −2.0 V for 120 s (Figure 4d). Similar coloration rate and transmittance modulation were observed for V^2+^-ECDs in the visible range (ΔTr = −40% at λ_abs_ = 608 nm), though it required the use of higher voltages (*E*_app_ = −2.5 V for 120 s) (Figure 4d). As expected, negligible NIR transmittance modulation (ΔTr = −4% at λ_abs_ = 845 nm) was measured for the V^2+^-based devices even at this higher potential, which demonstrates the superior capacity of (2V)^4+^-ECDs to control light transmission in the near-infrared region. Overall, the coloration efficiency determined for both types of ECDs in the visible region fell within the typical range for viologen-based ECDs,^24,28^ being slightly higher for the (2V)^4+^-based devices (166 cm^2^ C^–1^ for (2V)^4+^-ECDs and 146 cm^2^ C^–1^ for V^2+^-ECDs) (Figure 4e). After coloration, the bleached state of the ECDs was recovered by applying open circuit conditions for 180–240 s. A slightly slower bleaching kinetics was measured under these conditions for (2V)^4+^-ECDs, which can be attributed to the higher stability of the intramolecular pimer with respect to the monomeric V^2+^ radical cation.

Finally, the operation stability of both types of ECDs was investigated over multiple consecutive coloration-bleaching cycles (950 cycles, Figure 4f). Interestingly, the redox-induced transmittance modulation of (2V)^4+^-ECDs remained consistent over the whole cycling range, thus demonstrating that the formation of intramolecular pimers does not affect the stability of the electrochromic devices based on viologen tweezers. This is a very relevant result, as one of the principal mechanisms proposed for the lifespan shortening of ECDs based on monomeric viologens is radical ion pairing.^28^ In particular, it is considered that viologen aggregation takes place in these devices due to the lower solubility of the monomer radical cations V^•+^ generated upon reduction, which intermolecularly react to produce π-dimers that precipitate and eventually become electrochemically inactive. Notably, this process did not occur for the intramolecular pimers formed in our (2V)^4+^-ECDs, which might be explained by their higher solubility and stability that should lead to better electrochemical cyclability. In addition, (2V)^4+^-ECDs also showed high stability against different environmental factors, as they preserved their optimal electrochromic response after exposure to ambient humidity and illumination conditions for months or being subjected to heating and cooling treatments for several hours (Figure S7). In contrast, V^2+^-ECDs experienced a significant deterioration of their electrochromic response upon repetitive operation, especially during the first 150 cycles. We attribute this result to two main factors: the lower stability and solubility of the radical cations V^•+^ produced; and the higher reductive voltage required to obtain comparable coloration changes for V^2+^-ECDs (*E*_app_ = −2.5 V to reach ΔTr ∼ −40%), which could further accelerate the fatigue of the device by degrading the ITO working electrode or even the ionic liquid of the electrochromic IG layer.

### 2.3 Energy-Saving Performance of Viologen Tweezer-Based Smart Windows

The large electroinduced absorption changes exhibited by (2V)^4+^-based ECDs in both the visible and NIR regions make them very promising candidates for energy-saving ECWs. In their resting state, our 2V^4+^-ECDs show good transparency along the visible (400–750 nm, Tr_vis_^0V^ = 79.4%) and NIR ranges (750–1100 nm, Tr_NIR_^0V^ = 69.0%), in agreement with reference V^2+^-ECDs (Tr_vis_^0V^ = 79.3% and Tr_NIR_^0V^ = 68.1%) (Figure 5a,b). Therefore, the use of viologen tweezers instead of monomeric viologens as electrochromes in ECWs does not compromise sunlight transmittance in the bleached state, thus ensuring high solar illumination and heating indoors. In addition, (2V)^4+^-ECDs clearly overperform V^2+^-ECDs in terms of sunlight transmission modulation upon voltage application. Thus, when *E*_app_ = −2.0 V was applied for 400 s, a large variation in visible (Tr_vis_^–2V^ = 40%) and NIR light transmittance (Tr_NIR_^–2V^ = 33.4%) was measured for (2V)^4+^-ECDs, thus causing an efficient control over solar irradiation along these two spectral ranges (ΔTr_vis_^solar^ = −39.4% and ΔTr_NIR_^solar^ = −35.6%) (Figure 5a,b). On the contrary, for reference V^2+^-ECDs, a more reduced sunlight modulation effect (ΔTr_vis_^solar^ = −34% and ΔTr_NIR_^solar^ = −6%) was observed under equivalent operating conditions, even in the visible region (Figure 5a,b). Two main factors account for this situation. First, the absence (or very minor extent) of intermolecular pimerization in V^2+^-ECDs, which led to poor NIR absorption upon reduction, in contrast to (2V)^4+^-based devices. Second, the higher reduction potential of V^2+^ in comparison to (2V)^4+^, which made Δ*T*_vis_^solar^ also somewhat smaller for V^2+^-ECDs at *E*_app_ = −2.0 V. This effect was further proven by two additional experiments. On the one hand, we evaluated the absorption modulation of (2V)^4+^- and V^2+^-based devices at different voltages, which unambiguously proved that (2V)^4+^-ECDs produce more effective sunlight modulation at lower operating potentials such as *E*_app_ = −1.5 and −2.0 V (Figure 5c). Actually, a higher voltage (*E*_app_ = −2.5 V) was required to ensure a slightly larger variation in visible light transmittance with V^2+^-ECDs, though they still presented poor NIR modulation at this condition. On the other hand, we prepared a hybrid ECD with 0.125 wt % content of both (2V)^4+^ and V^2+^. Although separate reduction waves could be detected for (2V)^4+^ and V^2+^ in this hybrid device, its electrochromic response at low operating voltages (*E*_app_ = −1.5 and −2.0 V) was mainly controlled by pimerization (Figure S8). This can again be attributed to the lower reduction potential of (2V)^4+^, even though the occurrence of intermolecular (2V)^4+^–V^2+^ aggregation cannot be ruled out, which would further reduce the contribution of viologen monomers to the electrochromic response. Critically, this experiment also suggests that mixing (2V)^4+^ and V^2+^ in the same device neither spectrally broadens nor enhances the electrochromic behavior at low operation voltages.

**Figure 5:**
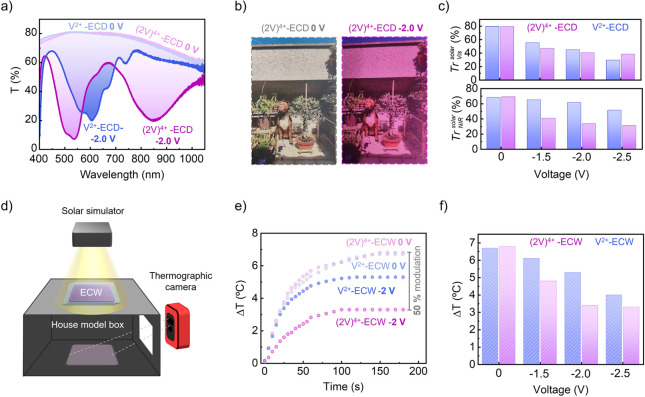
(a) Transmittance spectra of (2V)^4+^-ECDs (purple) and V^2+^-ECDs (blue) at *E*_app_ = 0 V and after applying *E*_app_ = −2.0 V for 400 s. (b) Images acquired across a (2V)^4+^-ECD at *E*_app_ = 0 and −2.0 V. (c) Solar transmittance of (2V)^4+^-ECDs and V^2+^-ECDs in the (top) visible region (400–750 nm) and (bottom) NIR region (750–1100 nm) under different voltages. (d) Schematic diagram of the setup used for the energy-saving tests. (e) Temperature variation inside the house model box during solar simulator irradiation through (2V)^4+^- and V^2+^-based ECWs at *E*_app_ = 0 and −2.0 V. (f) Temperature variations inside the house model after 3 min of irradiation with a solar simulator through (2V)^4+^- and V^2+^-based ECWs at different voltages.

The superior electrochromic performance of (2V)^4+^-based ECDs also rates well when compared with other types of NIR-responsive electrochromic systems reported in the literature (Figure S9).^34,74–81^ In terms of maximum transmittance modulation achieved in the NIR region (ΔTr_NIR_^max^), our system (ΔTr_NIR_^max^ ∼ 50%) lies just below the best cases reported (ΔTr_NIR_^max^ ∼ 60%) and performs clearly better than many of the organic-based NIR-responsive ECDs described to date (ΔTr_NIR_^max^ < 35%). More importantly, a large NIR transmittance modulation is achieved in our ECDs without detrimentally affecting the transparency of the initial state in the visible region, in contrast to most of the NIR-responsive electrochromes described, which significantly absorb in the blue region of the spectrum in the bleached state (Tr_425 nm_ < 70%). Thanks to our strategy exploiting intramolecular viologen pimerization, NIR transmittance modulation is reached in our system at relatively low voltages without the need of extending the π-conjugation of viologens, thus leading to negligible visible light absorption in the initial state of the device (Tr_425 nm_ ∼ 80%). As a result, our viologen dimer-based ECDs are one of the first cases described where redox-induced transition between true colorless and visible-/NIR-absorbing states is accomplished.

Prompted by the high capacity of (2V)^4+^-ECDs to regulate solar irradiation, we investigated their performance as ECWs. In these experiments, a (2V)^4+^-ECD (or a V^2+^-ECD as a reference) was used to cover a small aperture (2.5 × 2.5 cm) in a house model, and the temperature inside the system was monitored during irradiation with a solar simulator at an irradiance of 1 sun (100 mW cm^–2^ at AM 1.5 G condition) through the tested ECW (Figure 5d). As expected, the interior temperature evolved similarly for both types of ECWs in the bleached state: Δ*T*_2V^4+^‑ECD_^0V^ = 6.8 °C and ΔT_V^2+^‑ECD_^0V^ = 6.7 °C which indicates that they show similar transparency to sunlight in the absence of any voltage (Figure 5e). However, clearly different results were observed upon application of *E*_app_ = −2.0 V (Figure 5e). For the V^2+^-based ECW, redox-induced coloration led to a moderate reduction of solar heating (Δ*T*_V^2+^‑ECD_^–2V^ = 5.3 °C). By contrast, a much higher cooling effect was produced with the (2V)^4+^-containing ECW, which resulted in a remarkable 50% decrement in solar heat gain (Δ*T*_(2V)^4+^‑ECD_^–2.0V^ = 3.4 °C). The larger temperature reduction found for the (2V)^4+^-ECD should be mainly ascribed to higher modulation of the NIR component of sunlight (given by its ability to undergo intramolecular pimerization), enhancing the energy saving properties of smart windows. As a result, the superior performance of (2V)^4+^-ECDs as ECWs was maintained in other operating conditions and, thanks to the lower reduction potential of (2V)^4+^, maximum solar heating modulation could be accomplished at lower voltages than for V^2+^-based ECWs (Figure 5f). These results, together with the enhanced operation stability demonstrated above, prove that viologen tweezers provide several advantages for the fabrication of enhanced ECWs.

## 3 Conclusions

In this work we pioneered the rational use of supramolecular chemistry to enhance the performance of viologen-based electrochromic smart windows. For this, we took advantage of the intramolecular pimerization process of viologen dimers tethered through flexible linkers ((2V)^4+^), which lowers their reduction potential and allows extending the absorption spectra of the electroinduced viologen radical cations to the NIR region. Herein we demonstrated that such behavior is preserved in a variety of electrolytic media, including several ionic liquids and solid-state ionogels, at low concentrations, which allows preventing the detrimental effects on electrochromic fatigue resistance that result from the use of high viologen concentrations. In view of that, (2V)^4+^-based ionogels were applied to the construction of electrochromic devices, which exhibited several advantageous features over equivalent systems based on conventional viologen monomers (V^2+^): broader spectral response, lower operation voltage and higher stability, which could be achieved without compromising the electroinduced switching speed and coloration efficiency. As a consequence, when these (2V)^4+^-based devices were tested as electrochromic smart windows for energy saving, a 2.4-fold higher modulation of solar heat gain could be reached relative to V^2+^-based devices. Overall, these results demonstrate the potential of intramolecular viologen pimerization to improve their electrochromic performance for a variety of applications.

## 4 Experimental Section

### 4.1 Materials

4,4′-bipyridine, 1,3-diiodopropane, iodomethane, ammonium hexafluorophosphate salt (NH_4_PF_6_), ferrocene, lithium bis­(trifluoromethylsulfonyl)­imide (LiTFSI), tetrabutylammonium hexafluorophosphate (TBA PF_6_), Zn powder, poly­(vinylidene fluoride-*co*-hexafluoropropylene) (P­(VDF-*co*-HFP)), acetonitrile, diethyl ether and acetone were purchased from Sigma-Aldrich. All the ionic liquids used in this work were obtained from Solvionic (Toulouse, France). All reagents and compounds were used without further purification. Methyl viologen (V^2+^) chloride salt was purchased from Sigma-Aldrich and an ionic exchange with NH_4_PF_6_ was performed to obtain the V-2PF_6_ salt. The viologen dimer salt 2V-4PF_6_ was prepared as previously described (see the Supporting Information).^82^

### 4.2 Characterization Techniques

The electronic absorption of the compounds in acetonitrile solution were recorded on an Agilent Cary 5000 spectrophotometer with a PbSmart NIR detector. For the reduced species, samples were prepared by dissolving the compounds of interest in oxygen-degassed acetonitrile and mixing them with Zn powder in a glovebox. After 30 min under gentle stirring, the mixture was filtrated using 0.2 μm syringe filters and placed into a UV–vis cuvette with a septum cap. Then, the sample was taken out from the glovebox and the absorption spectra were immediately measured. The absorption spectra of the compounds in ionic liquids and ionogels was acquired using a Hamamatsu L10290 spectrophotometer. When needed, a potential was applied using a VSP100 potentiostat controlled by EC-Lab V9.51 software and coupled to the Hamamatsu L10290 spectrophotometer.

Electrochemical measurements in liquid solution (ACN/0.1 M TBA PF_6_ or ionic liquid) were performed with a VSP100 potentiostat in a conical cell using a 1 mm in diameter glassy carbon disk as a working electrode, a Pt disk (diameter <1 mm) as a counter electrode, and a standard Ag/AgCl reference electrode. The salt solution of the reference Ag/AgCl electrode was separated from the electrochemical solution by a salt-bridge ended with a frit, which was made of a ceramic material, allowing ionic conduction between the two solutions and avoiding appreciable contamination. The electrolyte solution present in the bridge was the same as the one used for the electrochemical solution, to minimize junction potentials. The error associated with the potential values is less than 5 mV. The ohmic drop can be one of the main sources of error when ILs are used as solvents, since they are more resistive media than aprotic polar solvents with 0.1 M concentration of supporting electrolyte. Spectroelectrochemical measurements in liquid solution (ACN/0.1 M TBA PF_6_ or ionic liquid) were performed in a 1 mm-optical path quartz cuvette using a Pt grid as a working electrode, a Pt wire as counter electrode and a Ag/AgCl as a reference electrode. All these experiments in solution were performed after degassing with an inert gas (N_2_ or Ar) for 10 min. Electrochemical and spectroelectrochemical measurements of the ionogels containing the viologen compounds were performed using screen-printed electrodes (SPE, DropSens), a three-electrode system composed of a carbon or an optically transparent ITO working electrode, a carbon counter electrode, and an Ag pseudoreference electrode.

The absorption and transmittance measurements of the electrochromic devices were obtained using an Agilent Cary 60 spectrophotometer, and a power supply was utilized to deliver the desired potential to the device. The stability of the electrochromic devices under 950 switching cycles was performed with the Hamamatsu L10290 spectrophotometer coupled to the VSP100 potentiostat controlled by EC-Lab V9.51 software. The energy-saving experiments were performed using an insulating polystyrene box as a house model with (i) a small aperture (2.5 cm × 2.5 cm) in the top covered with the electrochromic device, and (ii) a lateral aperture (5 cm × 5 cm) where the IR thermographic camera Uni-T UTi712S was placed to monitor the temperature. The box was irradiated through the small upper aperture using a solar simulator (Abet SunLite 11002, AM1.5G filter) at 100 mW cm^–2^.

The morphology of the IGs was characterized by means of confocal imaging as a noncontact optical 3D profiling technique using the DCM 3D optical profilometer (Leica) provided by the UAB Microscopy Service. AC impedance spectroscopy measurements were recorded using a 1287 potentiostat controlled by Zplot 3.5i software coupled with an electrochemical interface for frequency response analyzer (FRA, 1260A Impedance Analyzer).

The transmittance modulation in the solar (400–750 nm, %Tr_vis_) and NIR (750–1100 nm, %Tr_NIR_) ranges of the electrochromic devices was calculated as follows:

1 $$\%{\mathrm{Tr}}_{\mathrm{vis}/\mathrm{NIR}}=\frac{\int G\left(\lambda \right)\mathrm{Tr}\left(\lambda \right)d\lambda }{\int G\left(\lambda \right)d\lambda }\times 100$$

In eq 1, Tr­(λ) is the wavelength-dependent transmittance measured for the electrochromic devices and *G*(λ) is the solar irradiance spectrum ASTM G173–03 Global Tilted 37°.

### 4.3 Preparation of Ionogels

100 mg of P­(VDF-*co*-HFP), 500 mg of [BMIM]­[TFSI] (or [N_1114_]­[TFSI]) and 50 mg of LiTFSI were dissolved in 2.5 mL of acetone. The mixture was sonicated in an ultrasonic bath for 30 min at 30 °C to ensure dissolution of the polymer. To 1 mL of the obtained solution, 64 μL of a stock 25 mM (∼25.4 mg mL^–1^) solution of the viologen dimer (2V)^4+^ in acetonitrile were added. In this case, the final concentration of the viologen dimer in the final dry ionogel was calculated to be around 0.25 wt %. For the ionogels loaded with the monomeric methyl viologen V^2+^, 64 μL of a stock 50 mM (∼24.5 mg mL^–1^) solution of methyl viologen in acetonitrile were added to obtain a final concentration of 0.24 wt % on the dried ionogel. To prepare the ionogel, 180 μL (2 × 90 μL) of these mixtures were sequentially drop-casted (waiting for drying between one addition and the other) into a poly­(dimethylsiloxane) (PDMS) circular mold (diameter = 1 cm). The mixture was left to dry at room temperature overnight to obtain the free-standing ionogel. For the preparation of the ionogel containing 2.4 wt % of methyl viologen, 15.5 mg of methyl viologen were directly dissolved in 1 mL of the ionogel-acetone solution and the mixture was drop-casted and dried as the other samples.

### 4.4 Preparation of the Electrochromic Devices

To formulate the IG layer of the electrochromic devices, 100 mg of P­(VDF-*co*-HFP), 500 mg of the ionic liquid of interest, and 50 mg of LiTFSI salt were dissolved in 2.5 mL of acetone. Then, the mixture was sonicated in an ultrasonic bath for 30 min at 30 °C to ensure the dissolution of the polymer. To 1 mL of this solution, 64 μL of a 25 mM viologen dimer solution (or of a 50 mM viologen monomer solution, or of a 1:1 mixture of 12.5 mM viologen dimer and 25 mM viologen monomer solutions) and 138 μL of a 25 mM ferrocene solution in acetonitrile (4.6 mg mL^–1^) were finally added. To build up the device, a double-sided adhesive layer of 200 μm in thickness was used to create a 2.5 cm × 1 cm well on the top of a glass-ITO substrate. Then, 800 μL (4 × 200 μL) of the IG solution were drop-casted into the well, letting it dry for 20–30 min after each addition. After the last of these additions, the device was dried under air overnight. After that, the device was assembled into a sandwich structure using a second glass-ITO layer. The device was gently pressed to ensure good contact with the electrodes.

## Supplementary Material



## Notes

Additional data on the preparation and characterization of electrochromic materials and devices (PDF)

## Abbreviations

ECW: electrochromic smart windows
SW: smart windows
UV: ultraviolet
(NIR): near-infrared
(IL): ionic liquid
(BMIM): 1-butyl-3-methylimidazolium
(TFSI): bis­(trifluoromethylsulfonyl)­imide
(EMIM): 1-ethyl-3-methylimidazolium
(N_1114_): butyltrimethylammonium
IG: ionogel
P­(VDF-*co*-HFP): poly­(vinylidene fluoride-*co*-hexafluoropropylene)
TBA: tetrabutylammonium
Fc: ferrocene
ECD: electrochromic devices
ITO: indium tin oxide
PDMS: poly­(dimethylsiloxane).
