# Supplementary material for: Enhanced Electrochromic Smart Windows Based on Supramolecular Viologen Tweezers
Source: Chem Mater. 2025 Mar 12;37(6):2220–9. doi: 10.1021/acs.chemmater.4c03174 (PMC12124226; doi:10.1021/acs.chemmater.4c03174)
Supplement: Supplementary file 1 [file cm4c03174_si_001.pdf]

Supporting Information for:

Enhanced Electrochromic Smart Windows Based on Supramolecular Viologen Tweezers

Jaume Ramon Otaegui,[1] Silvia Mena,[1,2] Jovelt M. Dorsainvil,[3] Gonzalo Guirado,[1] Daniel Ruiz-Molina,[4] Jordi Hernando,[1,] Jonathan C. Barnes,[3,*] Claudio Roscini[4,]**

[1] Departament de Química, Universitat Autònoma de Barcelona, Edifici C/n, Campus UAB, 08193 Cerdanyola del Vallès, Spain. E-mail: jordi.hernando@uab.cat

[2] Instituto de Microelectrónica de Barcelona (IMB-CNM, CSIC), 08193 Cerdanyola del Vallès, Spain

[3] Department of Chemistry, Washington University in St. Louis, St. Louis, MO 63130 USA. E-mail: jcbarnes@wustl.edu

[4] Catalan Institute of Nanoscience and Nanotechnology (ICN2), CSIC and BIST, Campus UAB, Bellaterra, 08193 Barcelona, Spain. E-mail: claudio.roscini@icn2.cat

Content

Experimental section………………………………………….……………S2

Supplementary tables………………………………………..…..……….S3

Supplementary figures……………………………..……………..…S5

Bibliography……………………………………………………………...S13

Experimental section:

Synthesis of the 2V-4PF$_6$: Following an adapted procedure from the literature,[1] 600 mg (3.84 mmol) of 4,4′-bipyridine were mixed with 65 µL (166 mg, 0.64 mmol) of 1,3-diiodopropane in 10 mL of acetonitrile and heated up to 80 ºC under reflux overnight. Next, the crude was filtered, and the precipitate was washed with cold acetonitrile. Then, the precipitate was dissolved in hot water containing an excess of NH_4PF_6, and the precipitate was filtered and washed with cold water several times. After drying overnight, the solid was dissolved in 15 mL of acetonitrile at 50 ºC, and about 400 µL (908 mg, 6.4 mmol) of iodomethane were added to the mixture. The reaction was allowed to proceed overnight at 80 ºC under reflux. After cooling down the mixture, 5 mL of diethyl ether were added, and the mixture was filtered. The obtained powder was washed with cold acetonitrile, and after drying, it was dissolved in hot water and an excess of NH_4PF_6 was added. The precipitate was filtered and (2V)- 4PF$_6$ was obtained as an off-white solid (506.09 mg, 82 % yield). ^{1}H NMR (500 MHz, (CD$_3$CN): δ 8.95 (d, *J* = 5 Hz, 4H, 2xArH), 8.88 (d, *J* = 5 Hz, 4H, 2xArH), 8.48 (d, *J* = 5 Hz, 4H, 2xArH), 8.40 (d, *J* = 5 Hz, 4H, 2xArH), 4.79 (t, *J* = 7.5 Hz, 4H, 2xCH$_2$-Ar), 4.43 (s, 6H, 2xCH$_3$), 2.24 (m, 2H, CH$_2$) ppm.

Supplementary Tables:

Table S1: Absorption maxima of $V^{\bullet+}$ and $(2V)^{2\bullet+}$ in different solvents.[a]

Solvent	λ_{abs}^{max} $V^{\bullet+}$ (nm)	λ_{abs}^{max} $(2V)^{2\bullet+}$ (nm)
ACN/0.1 M TBA PF_6	608	534, 835
[BMIM][TFSI]	608	538, 840

[a] Determined by spectroelectrochemical measurements conducted with a Pt working electrode, a Pt counter electrode, and Ag/AgCl reference electrode. The reduced species $V^{\bullet+}$ and $(2V)^{2\bullet+}$ were produced by applying a controlled potential electrolysis at E_{app} = -0.75 V (vs Ag/AgCl) for several minutes.

Table S2: Standard reduction potentials of V^{2+} (10 mM) and $(2V)^{4+}$ (5 mM) in different solvents.[a,b]

IL	V-2PF_6^-		(2V)-4PF_6^-	
	E^0_1 (V vs Ag/AgCl)	E^0_2 (V vs Ag/AgCl)	E^0_1 (V vs Ag/AgCl)	E^0_2 (V vs Ag/AgCl)
acetonitrile	-0.449	-0.865	-0.334	-0.901
[BMIM] [TFSI]	-0.489	-0.896	-0.368	-0.939
[EMIM][TFSI]	-0.515	-0.914	-0.406	-0.973
$[N_{1114}]$[TFSI]	-0.479	-0.882	-0.371	-0.940
[BMIM][BF_4]	-0.378	-0.762	-0.267	-0.805
[BMIM][PF_6]	-0.445	-0.855	-0.342	-0.897

[a] Measured by cyclic voltammetry experiments in an electrochemical conical cell using a three-electrode system: 1-mm in diameter glassy carbon disk as working electrode, a platinum disk (diameter < 1 mm) as counter electrode, and Ag/AgCl reference electrode. Scan rate: 0.1 $V \cdot s^{-1}$. [b] $E_0 = (E_{pc} + E_{pa}) / 2$.

Table S3: Physical properties of [BMIM][TFSI]-based ionogels containing V-2PF_6 and (2V)-4PF_6.

IG	Transmittance (%)[a]	Thickness (μm)[b]	Conductivity (mS cm^{-1})[c]
V-2·PF_6^-	86.3 ± 15.8	254	0.168 ± 0.004
2V-4·PF_6^-	86.8 ± 10.8	263	0.198 ± 0.018

[a] Measured at λ = 550 nm. [b] Measured using an optical profilometer (see Figure S2). [c] Inverse of the resistivity (R_{IG}) obtained by fitting the Nyquist plot obtained through AC Impedance characterization (see Figure S1).

Supplementary Figures:

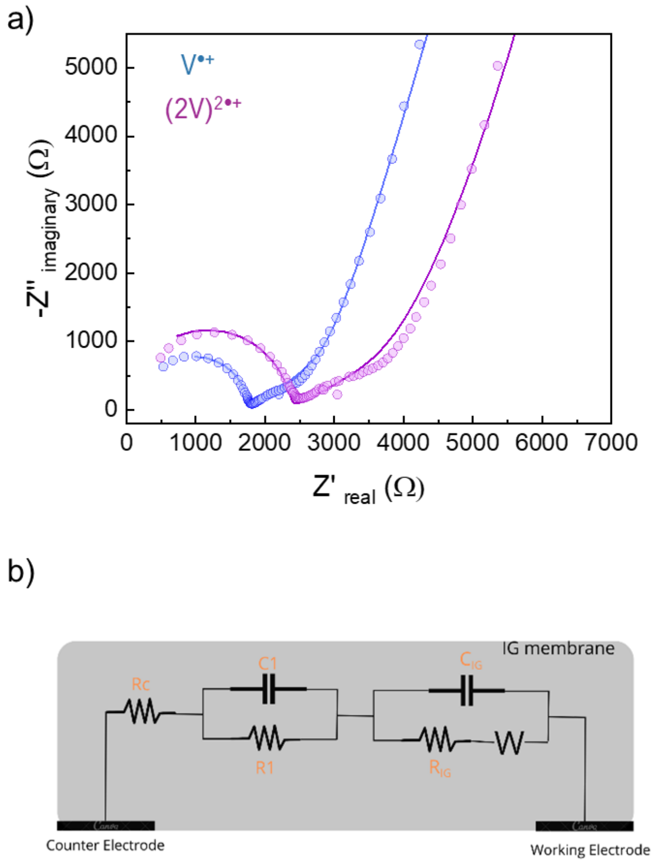

Figure S1: a) Impedance spectra (Nyquist plots) of [BMIM][TFSI]-based IGs containing $(2V)^{4+}$ and V^{2+}. Dots correspond to experimental data, while the lines are the fits to the circuit model shown in (b). b) Equivalent circuit model used to analyze the impedance spectra measured for the IGs in screen-printed electrodes.

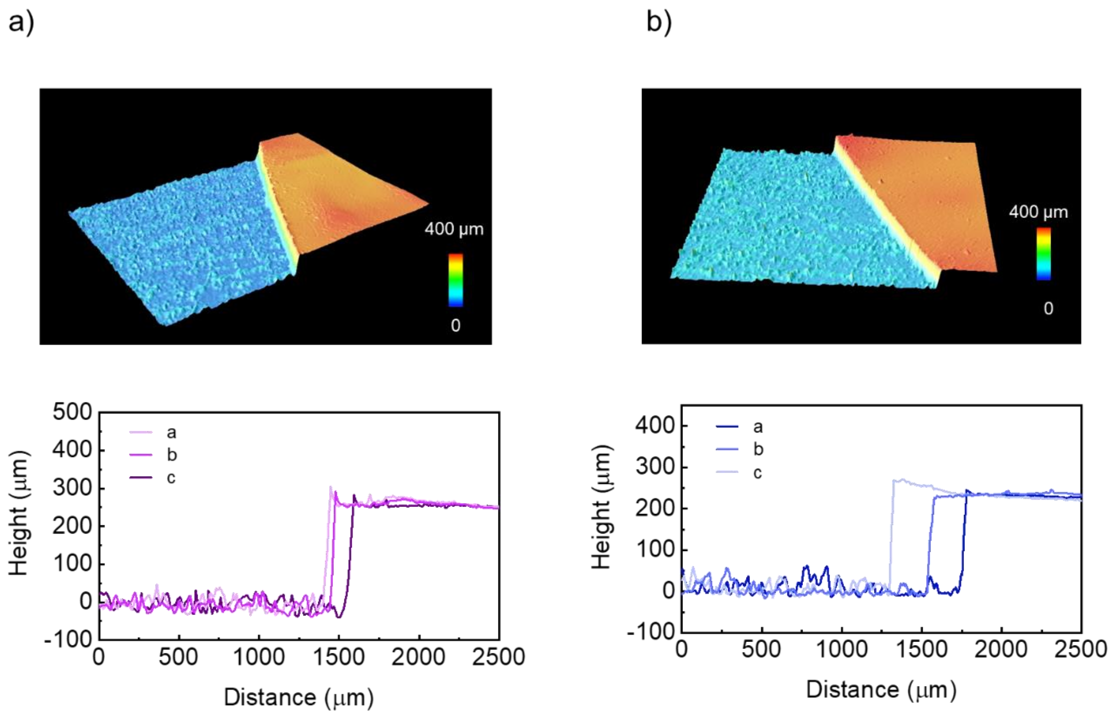

Figure S2: Optical profilometer characterization of [BMIM][TFSI]-based IGs containing (a) $(2V)^{4+}$ and (b) V^{2+}. Representative confocal stack images (top) and cross-section profiles of the membranes (bottom).

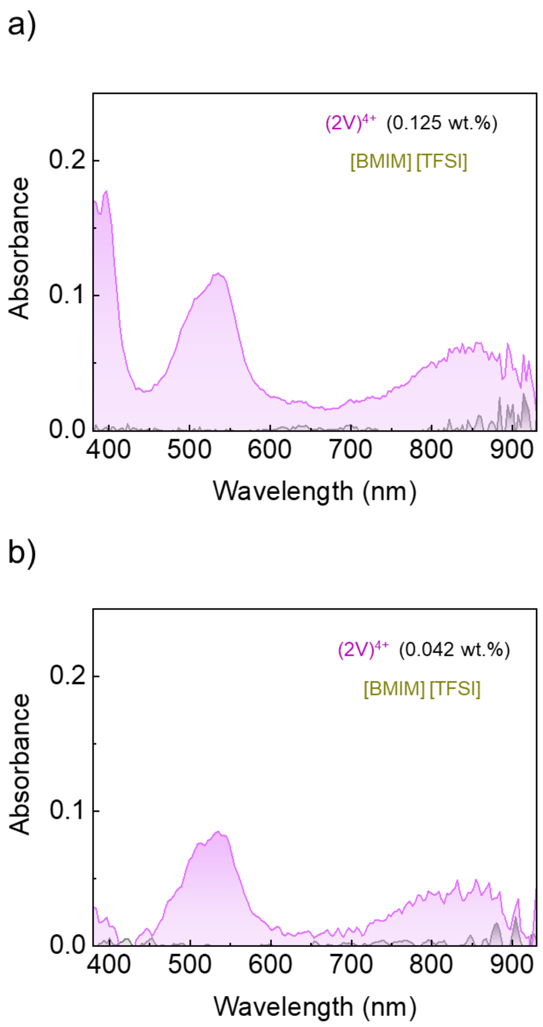

Figure S3: Absorption spectra of a [BMIM][TFSI]-based IG containing $(2V)^{4+}$ at a) 0.125 wt.% and b) 0.042 wt.% under 0 (gray) and -1.0 V (purple) of applied voltage for 60 s (working electrode: ITO; counter electrode: glassy carbon; pseudo-reference electrode: Ag). Attempts to further decrease $(2V)^{4+}$ concentration led to IGs showing negligible electrochromic responses both in the visible and NIR ranges.

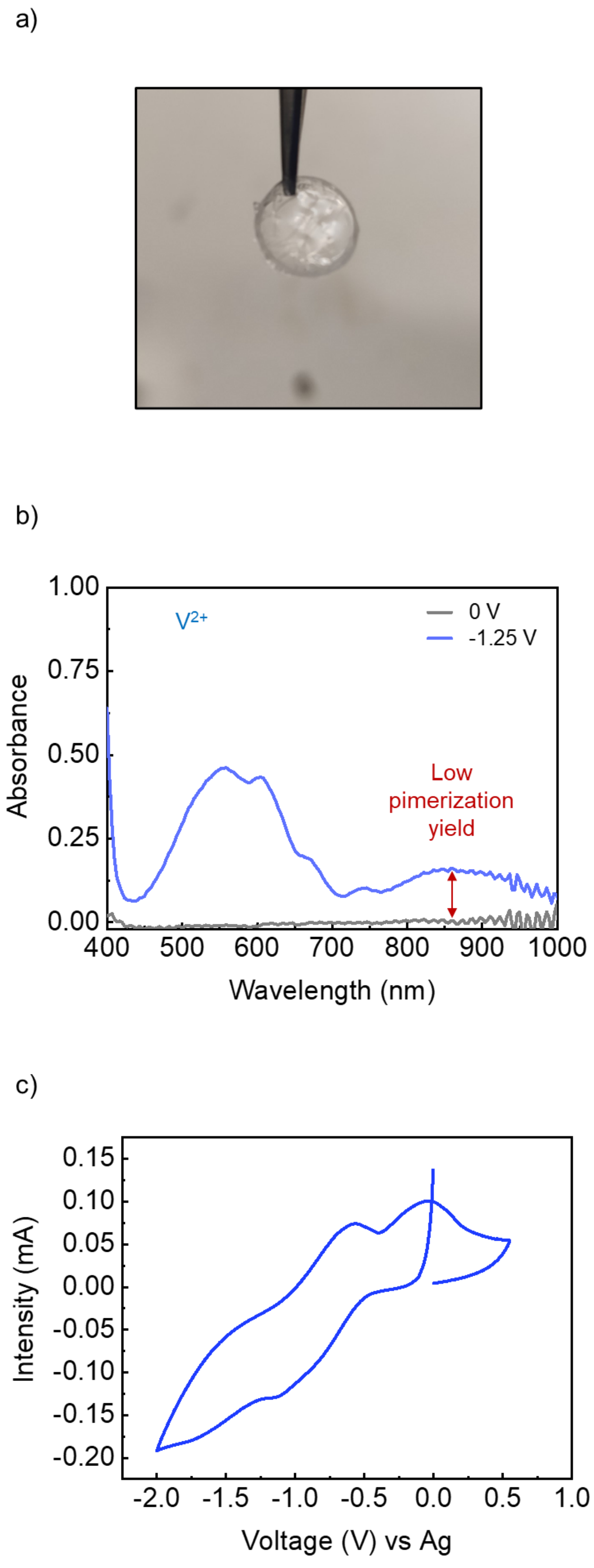

Figure S4: Characterization of a [BMIM][TFSI]-based IG containing 2.4 wt.% of V-$2PF_6$ (10 times more than the concentration used in this work for the preparation of electrochromic devices). a) Photograph of the obtained IG membrane with reduced transparency due to partial precipitation of the viologen salt. b)

Absorbance of the IG under 0 and -1.25 V of applied voltage for 60 s (working electrode: ITO, counter electrode: glassy carbon, pseudo-reference electrode: Ag). c) Cyclic voltammograms of the V^{2+}-based IG (2.4 wt.% V-2·PF_6) (working electrode: glassy carbon; counter electrode: glassy carbon; pseudo-reference electrode: Ag, scan rate: 0.1 V s^{-1}).

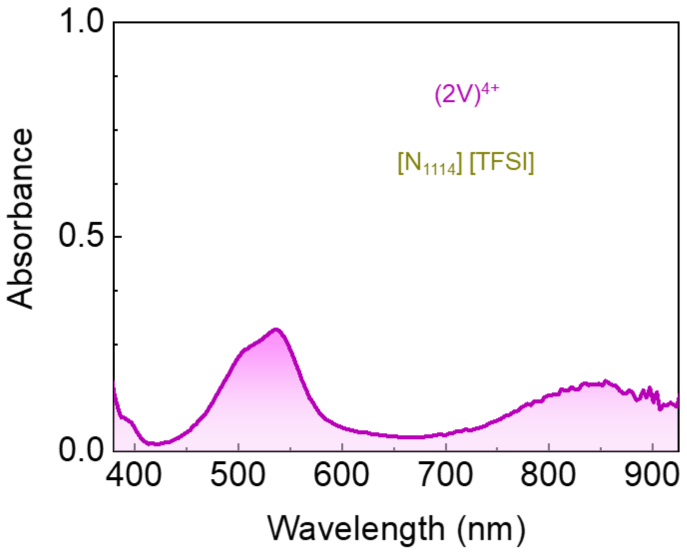

Figure S5: Absorption spectrum of a [N_{1114}][TFSI]-based IG containing (2V)4·PF_6 (0.25 wt.%) at -1.0 V of applied voltage for 60 s (working electrode: ITO; counter electrode: glassy carbon; pseudo-reference electrode: Ag).

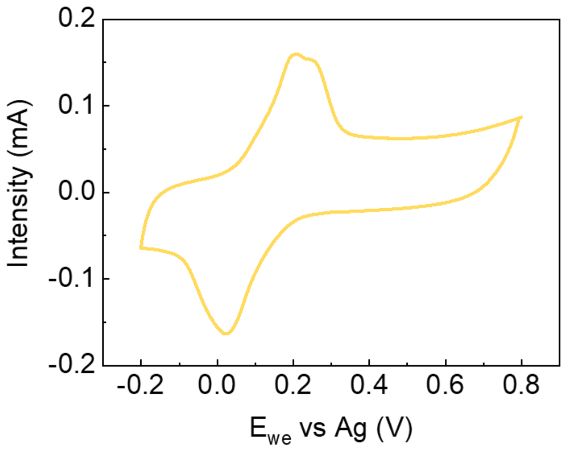

Figure S6: Cyclic voltammetry of a Fc-containing IG (0.1 wt.%) (working electrode: glassy carbon; counter electrode: glassy carbon; pseudo-reference electrode: Ag, scan rate: 0.1 V s^{-1}).

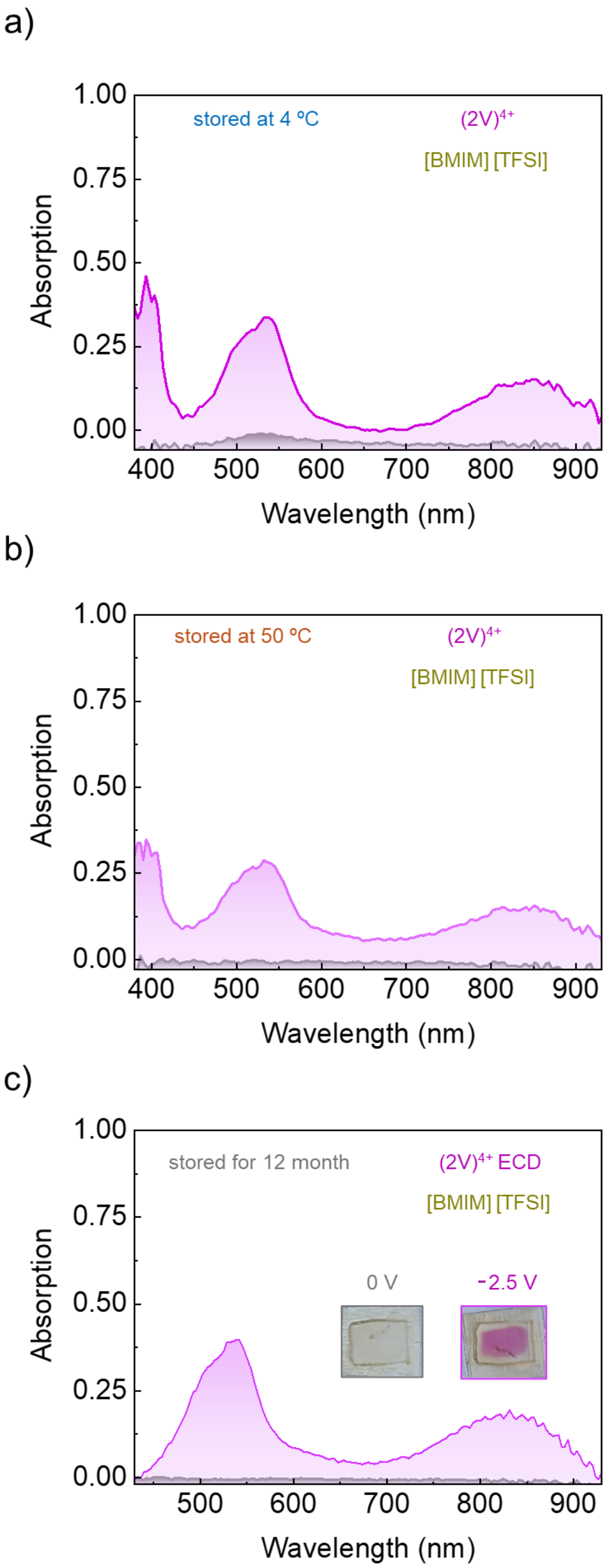

Figure S7: a-b) Absorption spectrum of a [BMIM][TFSI]-based IG containing (2V)4·PF_6 (0.25 wt.%) under 0 (gray) and -1.0 V (purple) of applied voltage for 60 s (working electrode: ITO; counter electrode: glassy carbon; pseudo-reference electrode: Ag) after a) 2 h storage at 4 ºC, and b) 2 h storage at 50 ºC. c) Absorption spectrum of a [BMIM][TFSI]-based ECD containing (2V)4·PF_6 (0.25 wt.%) after 12 months of exposure to ambient humidity and illumination conditions at 0 (gray) and -2.5 V (purple) of applied voltage for 60 s. The inset shows photographs of the device before and after application of -2.5 V.

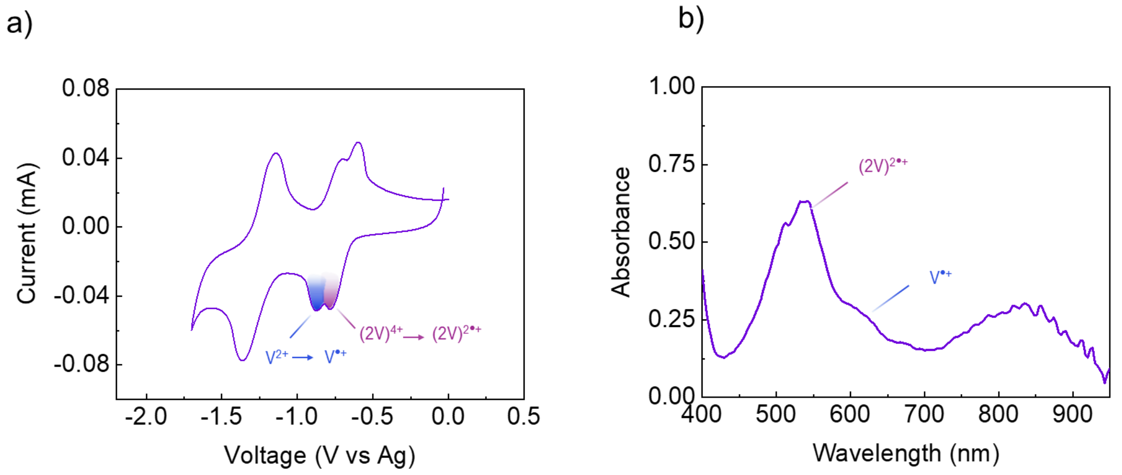

Figure S8: a) Cyclic voltammetry of a hybrid IG containing 0.125 % of $(2V)^{4+}$ and 0.124 % wt. of V^{2+} (1:1 molar ratio of viologen units) (working electrode: glassy carbon; counter electrode: glassy carbon; pseudo-reference electrode: Ag; scan rate: 0.2 V s^{-1}). The separate reduction waves of $(2V)^{4+}$ and V^{2+} are shown in the figure. b) Absorption spectrum of the same device after application of E_{app} =-1.8 V for 240 s. The spectral features in the visible range arising from the reduction of $(2V)^{4+}$ and V^{2+} are indicated in the figure.

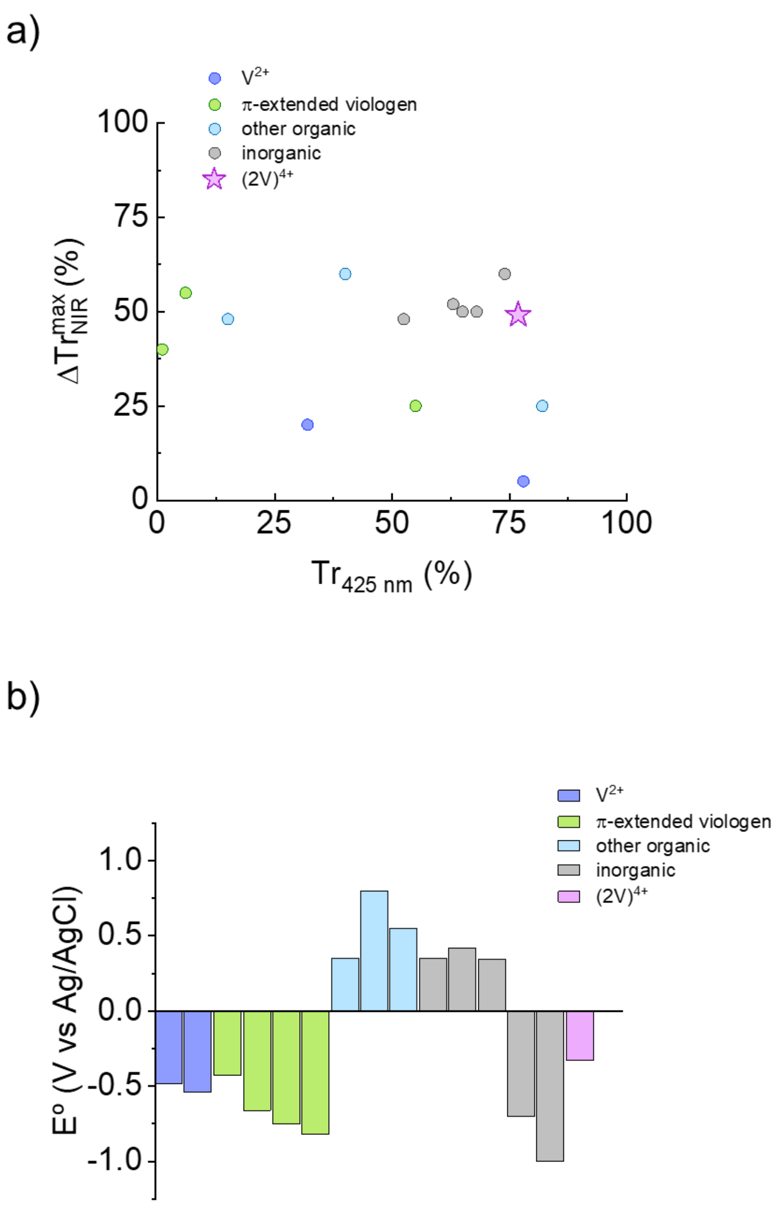

Figure S9: Comparison of the performance of different EC-based SWs with modulation in the NIR region. a) Plot of the maximum transmittance modulation achieved within the NIR spectral range vs the transmittance at 425 nm in the initial bleached state. Many ECWs that display significant modulation in the NIR region show an undesired yellowish color in their initial state because of non-negligible absorption within the spectral window 400-450 nm. In contrast, our $(2V)^{4+}$-based ECWs combine high transparency in the bleached state across all the visible spectrum with large NIR transmission modulation. b) Redox potentials (vs Ag/AgCl) of the different electrochromic materials utilized in previously reported NIR-responsive ECWs and of the viologen derivatives used in this work.[2–10]

References:

(1) Delawder, A. O.; Palmquist, M. S.; Dorsainvil, J. M.; Colley, N. D.; Saak, T. M.; Gruschka, M. C.; Li, X.; Li, L.; Zhang, Y.; Barnes, J. C. Iterative Step-Growth Synthesis and Degradation of Unimolecular Polyviologens under Mild Conditions. *Chem. Commun.* **2022**, *58*, 1358–1361. https://doi.org/10.1039/D1CC06912H.

(2) Wang, Z.; Jia, X.; Zhang, P.; Liu, Y.; Qi, H.; Zhang, P.; Kaiser, U.; Reineke, S.; Dong, R.; Feng, X. Viologen-Immobilized 2D Polymer Film Enabling Highly Efficient Electrochromic Device for Solar-Powered Smart Window. *Adv. Mater.* **2022**, *34*, 2106073. https://doi.org/10.1002/adma.202106073.

(3) Zhang, X.; Han, Z.; Cao, T.; Zhu, Y.; Wang, Z.; Zhang, Z.; Zhou, Z.; Zhang, D.; Yan, N.; Zhang, Y.; He, G. Multi-Responsive Thiazolothiazole Viologen-Based Electrochromic Materials for Smart Windows and Electronic Displays. *Chem. Eng. J.* **2025**, *503*, 158494. https://doi.org/10.1016/j.cej.2024.158494.

(4) Sun, F.; Zhang, H.; Cai, J.; Su, F.; Tian, Y.; Liu, Y. J. Selenophene, Thiophene, and Furan Functionalized π-Extended Viologen Derivatives for Tunable All-in-One ECDs. *Sol. Energy Mater. Sol. Cells* **2023**, *250*, 112106. https://doi.org/10.1016/j.solmat.2022.112106.

(5) Fu, X.; Li, K.; Zhang, C.; Wang, Q.; Xu, G.; Rogachev, A. A.; Yarmolenko, M. A.; Cao, H.; Zhang, H. Homogeneous and Nanogranular Prussian Blue to Enable Long-Term-Stable Electrochromic Devices. *ACS Appl. Mater. Interfaces* **2024**, *16*, 17745–17756. https://doi.org/10.1021/acsami.3c17551.

(6) Ma, D.; Lee-Sie Eh, A.; Cao, S.; Lee, P. S.; Wang, J. Wide-Spectrum Modulated Electrochromic Smart Windows Based on MnO2/PB Films. *ACS Appl. Mater. Interfaces* **2022**, *14*, 1443–1451. https://doi.org/10.1021/acsami.1c20011.

(7) Ganesha, M. K.; Mondal, I.; Singh, A. K.; Kulkarni, G. U. Fabrication of Large-Area, Affordable Dual-Function Electrochromic Smart Windows by Using a Hybrid Electrode Coated with an Oxygen-Deficient Tungsten Oxide Ultrathin Porous Film. *ACS Appl. Mater. Interfaces* **2023**, *15*, 19111–19120. https://doi.org/10.1021/acsami.2c22638.

(8) Zhang, S.; Peng, Y.; Zhao, J.; Fan, Z.; Ding, B.; Lee, J. Y.; Zhang, X.; Xuan, Y. Amorphous and Porous Tungsten Oxide Films for Fast-Switching Dual-Band Electrochromic Smart Windows. *Adv. Opt. Mater.* **2023**, *11*, 2202115. https://doi.org/10.1002/adom.202202115.

(9) Zhao, Y.; Liu, Q.; Wang, Y.; Liu, H.; Lv, M.; Cheng, P.; Fu, Y.; Li, J.; He, D. Smart Windows Built with a Conductive Polymer with Net Zero Energy Consumption. *Cell Rep. Phys. Sci.* **2022**, *3*, 101100. https://doi.org/10.1016/j.xcrp.2022.101100.

(10) Yilmaz, P.; Magni, M.; Martinez, S.; Gonzalez Gil, R. M.; Della Pirriera, M.; Manca, M. Spectrally Selective PANI/ITO Nanocomposite Electrodes for Energy-Efficient Dual Band

Electrochromic Windows. *ACS Appl. Energy Mater.* **2020**, *3*, 3779–3788. https://doi.org/10.1021/acsaem.0c00241.
